# Words to All

**Published:** 1864-10

**Authors:** W. W. Hall

**Affiliations:** New-York


### WORDS TO ALL.

We have been publishing the Journal of Health for these many years, and yet some of its readers have written to us from time to time, to know if we practised medicine. With such facilities as our monthly offers us for proclaiming our own praises, many a patientless doctor may have exclaimed in astonishment, "why don't you let the people know that you do give medical advice" ? We did not wish to engage in the general practice of medicine; we served our apprenticeship at that in the glorious sunshine of early youth; and naturally, when about turning the down hill of life, we wanted to take things easy, have a quiet mind and undisturbed nights; for the older we grow, the more we need quietude, evenness of life; rest for the body, calmness for the mind; this is the plan for any man who has reached fifty years to pave the way for a serene, cheery and healthful old age. How earnestly we wish that more men and women who have lived useful, should heed the idea so as to add another twenty-five years of good doing to the first fifty, instead of "giving out" at fifty-three or four or five as that lovely and lamented man, the good Dr. James W. Alexander under whose teachings we sat; and the Rev. R. S. Cook and many others. For twenty years we have given advice only to those who have sought it by letter or personally at our office. Within an hour we found the following letter on our office table among a goodly package from friends, kindred, patients and others.

"Dr. W. W. Hall, Dear sir, I called on you for advice on the eighth of January last and was under your care for six weeks ensuing, I am happy to say that my health has very much improved. I have not been in so good health for four years, as during the last six months. I have adhered to your directions as to diet, as closely as possible, and it has accomplished all that you promised me. My greatest trouble was with my LIVER. You gave me pills to act on that organ, and they were of great benefit to me. When I ceased taking them, I had one left. In June last, I had an attack of CATARRH, with slight soreness in the throat. I took the remaining pill and it cured me. About two weeks ago, I was attacked with CANKER in my mouth, it became very troublesome, and I have sought relief elsewhere in vain. For the last ten days I have been troubled very much with the CATARRH; there has been a constant discharge of THIN WATERY MATTER. I can breathe through my nose but a small part of the time. My tongue is coated with a THICK YELLOW MATTER when I wake in the morning, and sometimes it lasts during the day. I sleep well; before I was attacked with the canker, I was feeling better than for a long time and was gaining flesh. I have eaten but very little meat this summer, have not seemed to relish it. I eat loosening food, plenty of fruit and vegetables; have eaten a great deal of oat meal porridge. I have no cough, and have a grand appetite. My trouble seems to be SORE MOUTH and CATARRH. I used to have a great deal of PAIN IN MY SIDE, but that has left me entirely. I think that a pill or two like those you gave me before would do me good."

REPLY.—New-York, Sept. 8, 1864.—"Enclosed are five pills. The cause of the CANKER is in the LIVER and the pills will remove it. The dollar sent pays for the pills and another dollar will pay for the advice not to take them! Make them last as long as you can. I send them to keep, rather than to take. Have them by you for an emergency for SICK HEADACHE; FOUL TONGUE; ERUCTATIONS; SOUR STOMACH; DIARRHEA, DYSENTERY, CHOLERA, WANT OF STRENGTH and some other kindred symptoms which educated physicians know are one in origin. Although a pill removes CONSTIPATION, it also arrests LOOSENESS of the bowels until nature gathers force for a natural and healthful action. One pill ought usually to last a month or two or more. They should never be taken oftner than once a week, and nothing is needed to carry them off. I have never known them to take the patient off. Your own experience proves their ability to remove the ailments for which they are usually given. As the last five pills lasted you eight months, besides curing you of the things of which you complained, endeavor to make these second five last you as many years, as the need for them usually is at greater intervals, until they can be dispensed with altogether. One is usually taken on going to bed, and persons go about their business the next day without loss of time, but printed directions accompany them. You are not to give them to your friends. To those who have been my patients I furnish them as above, but strangers requiring advice for the first time, must send the usual fee of five dollars for the pills and the specific directions which each case requires.

"Surgical Tracts for the People," by H. A. Daniels, M. D., contains useful and interesting information in reference to all diseases of a surgical character such as deformities of the eye, nose, face, inflamed eyes, catarrh, piles, fistula, and various other ailments of the neighboring organs. Sent post paid by forwarding Twenty-Five cents to P. C. Godfrey, 831 Broadway, New-York.

EPILEPSY.—This intractable and unfortunate malady is said to be successfully treated in London by the alternate application of heat and cold to the spinal column; cannot some of our enterprising and accomplished American surgeons take up the practice?

CATARRH affecting the nose or head and oftener indicated by offensive discharges very mortifying to the patient, have been successfully treated by Dr. H. A Daniels, by a very ingenious, safe and effective mode of applying appropriate remedies to the parts affected; the cure is reported to have been accomplished in a very short time and to have been permanent; this will be good news to persons who have been long troubled in this manner. His office is at 10½ Union Square, New-York City.

FARM AND GARDEN.—A greenback well invested, is the expenditure of it in securing the American Agriculturist for the coming year. This is the opinion of Orange Judd, of fourteen hundred editors, and over one hundred thousand actual subscribers to that admirably conducted monthly publication, or from now to the end of 1865, fifteen numbers, for $1.15, if sent during October 1864.

"THE SEA AND LAND," published monthly for our gallant Jack Tars, at 50 cents a year, issued monthly at 52 John Street, New-York, by Rev'd Frank Jackson, a gentleman whom we believe to merit the confidence and generous patronage of every naval officer, of every ship owner, and of every man who is a friend to seamen and who desires their elevation and happiness.

We wish to know if the Presbyterian Church, Old School, has any place in New-York where their Tracts and Sunday School books are sold, and if their establishment is "closed up" for want of patronage. There are a good many persons who read this Journal who would prefer to have their publications, and we for one don't know whether they are doing anything now or not. If they are, and will give us information in writing, or send us a list of their books, or some specimens of handiwork, we will report in the November number. But we certainly are sorry to see their indifference towards having their issues made known through every channel which could convey the knowledge of them to large numbers of readers. Are the officers paid by the day, like our street sweepers and government employees, of whom so many care but for one thing—to get their salaries promptly and to the uttermost farthing. Wake up you old rip-van-winkles! the fields are white, the time is short, and you will soon be "wanted," above or below, can't say which.

The Editor of Hall's Journal of Health will be found daily at the Publication office No. 12 Union Square, New-York, east side opposite the Monument, near Fourteenth Street. Office hours from 10 to 2 daily, and often at other hours. Address all letters to "DR. W. W. HALL, New-York."

